# Draft Genome Sequence of *Bacillus toyonensis* VU-DES13, Isolated from *Folsomia candida* (Collembola: Entomobryidae)

**DOI:** 10.1128/genomeA.00287-17

**Published:** 2017-05-11

**Authors:** Thierry K. S. Janssens, Tjalf E. de Boer, Valeria Agamennone, Niels Zaagman, Nico M. van Straalen, Dick Roelofs

**Affiliations:** a Department of Ecological Science, Vrije Universiteit Amsterdam, Amsterdam, The Netherlands; b MicroLife Solutions, Amsterdam, The Netherlands

## Abstract

We present here the draft genome of *Bacillus toyonensis* VU-DES13, which was isolated from the midgut of the soil-living springtail *Folsomia candida*. Previous research revealed the presence of gene clusters for the biosynthesis of various secondary metabolites, including β-lactam antibiotics, in the host's genome. The genome data are discussed in the light of the antimicrobial properties against fungi and oomycetes and a high level of β-lactam resistance of the isolate.

## GENOME ANNOUNCEMENT

*Bacillus toyonensis* strain VU-DES13 was isolated from the midgut of the soil-dwelling springtail *Folsomia candida*, which displays resistance to and can thrive on entomopathogens (1). The host’s genome contains biosynthetic gene clusters for secondary metabolites, such as for β-lactams (2), which are induced in the gut epithelium upon stress (3). Previous amplicon-sequencing studies (4, 5) revealed the prominence of *B. cereus* in the *F. candida* midgut. An association between the propagation cycle of members of the *B. cereus* clade and the internal environment of animals has been suggested (6, 7).

In this study, we provide the draft genome of the isolate *B. toyonensis* VU-DES13, which exhibited antimicrobial properties against fungi and oomycetes and a high level of β-lactam resistance in a MIC assay (>800 μg/mL penicillin G). We hypothesize that this microorganism represents a key player in colonization resistance to entomopathogens selected upon by the host.

High-molecular-weight gDNA was extracted from an overnight culture with the Macherey-Nagel Nucleospin soil kit. The genomic library was made by enzymatic shearing with the Ion Xpress Plus fragment library kit (Thermo Fisher) and size selection on a 2% agarose E-Gel SizeSelect Gel (Thermo Fisher). The template was prepared with 10 pM of the library on an Ion One Touch 2 system (Thermo Fisher). A 400-bp run was executed on an Ion Torrent PGM sequencer, with the Ion PGM Hi-Q sequencing kit (Thermo Fisher). After removal of adapters, the sequences were assembled with SPAdes (8). The assembly was annotated with the Prokka pipeline (9). Biosynthetic gene clusters for secondary metabolites, plasmid-related elements, and open reading frames (ORFs) related to antibiotic resistance were screened by anti-SMASH version 3.0.5 (10), PlasmidFinder version 1.3 (11), and ResFam (12). Feature frequency profiling (13) and genome BLAST distance phylogeny (14) were performed against representative *B. cereus* clade genomes in order to the determine the phylogeny of this isolate, which was previously identified by 16S rRNA sequencing as belonging to this clade.

The draft genome consisted of 5.45 Mb (35%GC), in 40 contigs, with 119× average coverage, an *N*_50_ of 254 kb, and an *L*_50_ of 9. Moreover, 5,512 ORFs, 53 tRNAs, 6 rRNA clusters, and 10 biosynthetic gene clusters for secondary metabolites were predicted. We were not able to confirm the presence of any plasmid, although seven contigs exhibited BLASTn hits with a number of *Bacillus* plasmids. Our isolate was positioned within *B. toyonensis*, of which the type strain BCT-7112, isolated from soil, is used as a feed supplement (15). Compared to BCT-7112^T^, VU-DES13 exhibited hits for three extra biosynthetic gene clusters by antiSMASH, as well as 13 additional β-lactamases. No virulence genes related to other *B. cereus* clade members (*B. anthracis*, *B. cereus*, and *B. thuringiensis*) were observed.

### Accession number(s).

Genome sequence data have been deposited in GenBank under accession number MWMG00000000.
